# Cell-Laden Agarose-Collagen Composite Hydrogels for Mechanotransduction Studies

**DOI:** 10.3389/fbioe.2020.00346

**Published:** 2020-04-21

**Authors:** Elena Cambria, Silvio Brunner, Sally Heusser, Philipp Fisch, Wolfgang Hitzl, Stephen J. Ferguson, Karin Wuertz-Kozak

**Affiliations:** ^1^Institute for Biomechanics, ETH Zurich, Zurich, Switzerland; ^2^Research Office (Biostatistics), Paracelsus Medical University, Salzburg, Austria; ^3^Department of Ophthalmology and Optometry, Paracelsus Medical University, Salzburg, Austria; ^4^Research Program Experimental Ophthalmology and Glaucoma Research, Paracelsus Medical University, Salzburg, Austria; ^5^Department of Biomedical Engineering, Rochester Institute of Technology, Rochester, NY, United States; ^6^Spine Center, Schön Klinik München Harlaching, Academic Teaching Hospital and Spine Research Institute of the Paracelsus Private Medical University Salzburg (Austria), Munich, Germany

**Keywords:** blended hydrogels, agarose, collagen, mechanobiology, extracellular matrix, dynamic compression, focal adhesion kinase

## Abstract

The increasing investigation of cellular mechanotransduction mechanisms requires biomaterials combining biofunctionality and suitable mechanical properties. Agarose is a standard biomaterial for cartilage and intervertebral disc mechanobiology studies, but lacks adhesion motifs and the necessary cell-matrix interaction for mechanotransduction. Here, collagen type I was blended at two concentrations (2 and 4.5 mg/mL) with agarose 2% wt/vol. The composite hydrogels were characterized in terms of structural homogeneity, rheological properties and size stability. Nucleus pulposus (NP) cell viability, proliferation, morphology, gene expression, GAG production, adhesion and mechanotransduction ability were further tested. Blended hydrogels presented a homogenous network of the two polymers. While the addition of 4.5 mg/mL collagen significantly decreased the storage modulus and increased the loss modulus of the gels, blended gels containing 2 mg/mL collagen displayed similar mechanical properties to agarose. Hydrogel size was conserved over 21 days for all agarose-based gels. Embedded cells were viable (>80%) and presented reduced proliferation and a round morphology typical of NP cells *in vivo*. Gene expression of collagen types I and II and aggrecan significantly increased in blended hydrogels from day 1 to 7, further resulting in a significantly superior GAG/DNA ratio compared to agarose gels at day 7. Agarose-collagen hydrogels not only promoted cell adhesion, contrary to agarose gels, but also showed a 5.36-fold higher focal adhesion kinase phosphorylation (pFAK/β-tubulin) when not compressed, and increased pFAK/FAK values 10 min after compression. Agarose-collagen thus outperforms agarose, mimics native tissues constituted of non-fibrillar matrix and collagens, and allows exploring complex loading in a highly reproducible system.

## Introduction

As many degenerative and regenerative processes are related to mechanical loading, the field of mechanobiology is widely expanding. The interest in understanding how mechanical signals influence cell behavior is in fact shared across disciplines as diverse as cancer and developmental biology, regenerative medicine, tissue engineering, and clinical disciplines such as pulmonology and orthopedics (Jaalouk and Lammerding, 2009; Martino et al., 2018; Miller et al., 2020). As an example, mechanical loading is a known contributor to intervertebral disc (IVD) degeneration, which when associated with pain and inflammation, is defined as degenerative disc disease (Adams and Roughley, 2006). Degenerative disc disease is a leading source of low back pain, which is itself the first cause of disability worldwide with a lifetime prevalence of 84% (Balague et al., 2012).

While physiological mechanical loading promotes the metabolic balance of the IVD (Chan et al., 2013), hyperphysiological loading provokes cell catabolism, inflammation, and reduced cell viability (Chan et al., 2011). Partly due to poor knowledge about the underlying molecular mechanisms, current treatments, such as anti-inflammatory drugs, are not targeted and have low effect sizes (Balague et al., 2012). Therefore, studies aiming at unlocking mechanotransduction mechanisms in the IVD are needed.

Mechanotransduction *in vivo* takes place in a three-dimensional (3D) microenvironment with instructive biochemical cues and specific mechanical stimuli (Martino et al., 2018). Conducting mechanotransduction studies with *in vivo* relevance thus requires advanced 3D culture systems that possess both the biofunctionality of native extracellular matrix (ECM) proteins, and tunable mechanical properties. However, such biomaterials are rare. Agarose has established itself as a gold standard biomaterial for dynamic compression studies, primarily in the field of cartilage tissue engineering (Anderson and Johnstone, 2017). Agarose is a linear polysaccharide derived from red algae and consisting of β-1,3-linked-D-galactose and α-1,4-linked 3,6-anhydro-L-galactose units (Velasco et al., 2012). The gelling mechanism of agarose resides in the formation and aggregation of double helices by intermolecular hydrogen-bonds upon cooling (Velasco et al., 2012). Agarose offers biocompatibility, retention of round cell morphology, homogeneity and strong mechanical properties (Bougault et al., 2009). The elastic modulus of agarose ranges from ∼1 to a few 1000 kPa, depending on polymer concentration and molecular weight (Normand et al., 2000). However, agarose is bio-inert and does not present any cell adhesion motifs. This characteristic is a shortcoming in the investigation of mechanotransduction, where most of the mechanisms are thought to originate from the ability of mechanosensors, such as integrins, to interact with the surrounding ECM (Lee et al., 2019). In fact, the positive effects of mechanical loading on cell-laden agarose constructs seem to become apparent only after a pre-culture period aiming to increase pericelluar matrix production (Anderson and Johnstone, 2017).

To enhance the biofunctionality of agarose, covalent modifications with adhesive peptides or proteins have been achieved through 1’1, carbonyldiimidazole (CDI) chemistry (Bellamkonda et al., 1995; Borkenhagen et al., 1998; Yu et al., 1999), reaction with S-2-nitrobenzyl cysteine (S-NBC) (Luo and Shoichet, 2004), crosslinking with sulfosuccinimidyl 6-(4′-azido-2′-nitrophenylamino)hexanoate (sulfo-SANPAH) (Dodla and Bellamkonda, 2006; Connelly et al., 2008, 2011; Au et al., 2012; Schuh et al., 2012), and carboxylation and EDC (1-ethyl-3-(3-dimethylaminopropyl) carbodiimide) condensation (Su et al., 2013). Interestingly, in a 3D covalently crosslinked collagen type IV-agarose system, the effect of high rate shear deformation aiming to mimic traumatic injury on neurons was enhanced by increasing collagen concentration (Cullen et al., 2007). Nevertheless, covalent modifications are time-consuming and involve cytotoxic reagents that need to be extensively washed out.

In order to improve the mechanical properties of natural ECM protein hydrogels, it is possible to physically blend-in agarose at low concentrations (Ulrich et al., 2010; Lake and Barocas, 2011; Lake et al., 2011). Ulrich et al. found that adding agarose into collagen I 3D hydrogels largely increased their elasticity and reduced cell migration (Ulrich et al., 2010). Similar collagen-agarose co-gels were mechanically tested under uniaxial tension (Lake and Barocas, 2011) and indentation (Lake et al., 2011). Conversely, and as an alternative to covalent modifications, the polymer blending technique can also be used to incorporate peptides and proteins in agarose to improve its bioactivity. Yamada and colleagues have blended laminin active peptides in 2D agarose gels and have shown enhanced cell adhesion based on substrate stiffness (Yamada et al., 2012). Composite agarose-based microbeads blended with collagen or fibrinogen/hydroxyapatite have been produced by emulsification (Batorsky et al., 2005; Rioja et al., 2017) and used for osteogenic differentiation (Lund et al., 2008) and vasculogenesis (Rioja et al., 2017), respectively. Nevertheless, the concentrations of agarose used in these studies were relatively low (up to 1% wt/vol), while the typical concentration to enable suitable mechanical stability and load transmission in dynamic compression studies is equal or above 2% wt/vol (Anderson and Johnstone, 2017). In this study, the final agarose concentration was kept constant to 2% wt/vol and collagen I was physically blended at two final concentrations of 2 and 4.5 mg/mL.

We aimed to develop novel agarose-collagen composite hydrogels that: (i) simultaneously combine the mechanical qualities of 2% wt/vol agarose and the biofunctionality of collagen I; (ii) mimic native tissues constituted of non-fibrillar matrix and collagens; and (iii) allow the investigation of complex loading and cellular mechanotransduction. To this end, we characterized hydrogels in terms of homogeneity, mechanical properties, size stability, and assessed their effect on IVD cell viability, proliferation, gene expression and glycosaminoglycans (GAG) content. Furthermore, we tested the hypothesis that agarose-collagen hydrogels display enhanced cell adhesion and mechanotransduction capacity compared to agarose hydrogels, as measured by focal adhesion kinase (FAK) phosphorylation after dynamic compression.

## Materials and Methods

### Cell Isolation and Culture

Tails from 18 to 24-month-old cows were obtained from the local slaughterhouse and carefully dissected to expose the IVDs. Nucleus pulposus (NP) biopsies were minced and digested overnight at 37°C, 5% CO_2_, using 0.4% collagenase NB4 (17454.01, Serva) and 0.2% dispase II (04942078001, Roche) dissolved in PBS 1X with 5% Antibiotic-Antimycotic (Anti-Anti; 15240-062, Gibco). The next day, the tissue digest was filtered through a cell strainer and the cell suspension was centrifuged at 1000 rpm for 5 min. The pellet was washed with culture medium (Dulbecco’s modified Eagle medium/F-12 Nutrient Mixture (DMEM/F12; 31330-038, Gibco), 10% fetal calf serum (FCS; F7524, Sigma-Aldrich) and 1% Anti-Anti). After centrifugation and resuspension in culture medium, cells were seeded in fibronectin-coated (0.01 mg/mL human plasma fibronectin; FC010, Merck Millipore) culture flasks (150 cm^2^). Cells were expanded to passage 1–2 at 37°C, 5% CO_2_, and the medium was changed twice per week.

### Hydrogel Fabrication and Culture

#### Agarose Hydrogels

Agarose 4% wt/vol (1.6 g agarose powder; 50101, SeaPlaque low-gelling temperature, Lonza; dissolved in 40 mL PBS 1X and autoclaved at 120°C) was first heated in the microwave and cooled down to 40°C. Cells were trypsinized and resuspended at a density of 8 × 10^6^ cells/mL in PBS 1X at 4°C. The agarose solution was then mixed 1:1 with the cell suspension and vortexed to obtain a final cell concentration of 4 × 10^6^ cells/mL in agarose 2% wt/vol. Using a positive displacement pipette, 160 μL of the mixture were molded in a silicon ring (inner Ø: 8 mm, outer Ø: 12 mm, height: 3 mm) placed between two microscope glass slides. Agarose hydrogels gelled within 1–2 min at room temperature (RT).

#### Collagen Hydrogels

Bovine collagen I (6 mg/mL; 5010, Advanced BioMatrix) was mixed on ice with UltraPure Distilled Water (Invitrogen), PBS 10X and 1M NaOH according to the manufacturer protocol to obtain a 5 mg/mL collagen solution. Pelleted cells were resuspended and vortexed with the collagen solution to obtain a concentration of 4 × 10^6^ cells/mL. Hydrogels were molded as described above and incubated for 30 min at 37°C until complete gelation.

#### Agarose-Collagen Hydrogels

Agarose 4% wt/vol was heated and kept at 60°C. In parallel, bovine collagen I (10 mg/mL; 5133, Advanced BioMatrix) was mixed with UltraPure Distilled Water according to the manufacturer protocol to obtain a 9 or 4 mg/mL collagen solution. Pelleted cells were resuspended in the collagen solution to obtain an 8 × 10^6^ cells/mL suspension. The solution was then mixed 1:1 and vortexed with the agarose 4% wt/vol solution to obtain a final concentration of 4 × 10^6^ cells/mL in agarose 2% wt/vol and collagen 4.5 or 2 mg/mL. Hydrogels were molded as described above, incubated for 10 min at RT, and for 30 min at 37°C until complete gelation.

Hydrogels were demolded and cultured in 12-well plates in culture medium at 37°C, 5% CO_2_ for up to 21 days. The medium was changed every other day.

### Confocal Reflection Microscopy of Collagen I

One day after hydrogel fabrication, cell-free hydrogels were incubated for 1 h in PBS 1X and imaged with a confocal microscope (Leica TCS SP8). Images were acquired in the reflection mode with a 488 nm laser and a 25X water objective. The reflected light was detected in the range from 475 to 795 nm. Images were stitched in order to image a whole gel.

### Rheometry

Oscillatory measurements were performed on an Anton Paar MCR301 Rheometer (Anton Paar, Switzerland), equipped with a 20 mm diameter parallel plate geometry (PP20; Anton Paar, Switzerland) and a peltier element with hood (H-PTD 200; Anton Paar, Switzerland). To avoid slipping of the hydrogels during oscillation, the plate and the probe of the rheometer were coated with 330 μL 0.1 mg/mL PLL at 60°C. A wet tissue was placed inside the hood to avoid evaporation of the hydrogels. Rheometry was performed with cell-free hydrogels at a gap of 1 mm, with a deflection angle of 1% and a frequency of 1 Hz. The measurement was performed in several intervals with temperatures ranging from 24 to 37°C depending on the hydrogel type (Table 1).

**TABLE 1:** Duration and temperature of rheometry intervals depending on hydrogel type.

**Gel**	**Interval 1**	**Interval 2**	**Interval 3**	**Interval 4**	**Interval 5**	**Interval 6**
Agarose	3.6 min 33°C→24°C	240 min 24°C	5.3 min 24°C→37°C	210 min 37°C	5.3 min 27°C→24°C	210 min 24°C
Agarose-collagen	3.6 min 33°C→24°C	300 min 24°C	5.3 min 24°C→37°C	270 min 37°C	5.3 min 27°C→24°C	270 min 24°C
Collagen	10 min 13°C→37°C	240 min 37°C	5.3 min 37°C→24°C	215.3 min 24°C		

### Hydrogel Dimension Measurement

At days 1, 7 and 21 after fabrication, cell-laden hydrogels were placed under a camera positioned at constant height. Pictures were acquired and the hydrogel diameter was evaluated with the distance measurement function of the ImageJ software. The height of the hydrogels was measured with a mechanical tester (TA.XT*plus* Texture Analyzer, Stable Micro System), with a 5 kg load cell.

### Viability Assay

At days 1, 7 and 21 after fabrication, hydrogels were incubated in NucBlue Live Cell Stain (R37605, Thermo Fisher Scientific) and 2 μM ethidium homodimer (46043, Sigma-Aldrich) in culture medium for 1 h at 37°C in the dark. Hydrogels were washed with PBS 1X and imaged with a confocal microscope (Leica TCS SP8) and a 20X dry objective. Six images per gel from 20 to 120 μm from the bottom of the gel (Z-stack) were acquired. Automated cell counting based on particle size and circularity was performed for each image using the ImageJ software. The cell viability percentage was calculated as the difference between the number of NucBlue-stained cells and the number of ethidium homodimer-stained cells, divided by the number of NucBlue-stained cells.

### Proliferation Assay

At days 1, 7 and 21 after fabrication, hydrogels were incubated for 24 h in 1 μM EdU in culture medium at 37°C (Click-iT EdU Imaging Kit Alexa-Fluor 488, C10337, Thermo Fisher Scientific) and washed 3 × 5 min with PBS 1X. Hydrogels were then fixed with 4% formaldehyde for 30 min at RT and washed 3 × 5 min with blocking solution (3% BSA (A6588, AppliChem) in PBS 1X). Cells were permeabilized with 0.25% Triton X-100 (A4975, AppliChem) in blocking solution for 30 min and hydrogels were washed 3 × 5 min with blocking solution. The reagents of the kit were mixed according to the manufacturer protocol except for the Alexa Fluor azide, which was used at half of the recommended concentration. Hydrogels were incubated for 45 min in Click-IT reaction cocktail in the dark, washed three times with blocking solution, and incubated for 30 min in 2 μg/mL DAPI (62248, Thermo Fisher Scientific) in PBS 1X. After 3 × 5 min washes in PBS 1X, hydrogels were imaged with a confocal microscope (Leica TCS SP8), using a 20X dry objective and a 488 nm laser to visualize the proliferating cells. Automated cell counting based on particle size and circularity was performed using the ImageJ software. The proliferating cell percentage was calculated as the number of Alexa-Fluor 488-stained cells divided by the number of DAPI-stained cells.

### 3D Cell Morphology Imaging

At days 1, 7 and 21 after fabrication, hydrogels were washed three times with PBS 1X and fixed with 4% formaldehyde (252549, Sigma-Aldrich) for 30 min at RT. After incubation in blocking solution (3% BSA, 0.5% TWEEN 20 (P1379, Sigma-Aldrich) in PBS 1X) for 3 × 5 min, cells were permeabilized with 0.25% Triton X-100 in blocking solution for 30 min. Hydrogels were washed for 3 × 5 min with blocking solution and then incubated in the dark for 2 h in 6.6 nM Alexa Fluor 568 phalloidin (A12380, Thermo Fisher Scientific) in blocking solution. After three washes in blocking solution, hydrogels were incubated for 30 min in 2 μg/mL DAPI diluted in PBS 1X. Hydrogels were finally washed 3 × 5 min in PBS 1X and imaged with a confocal microscope (Leica TCS SP8), using a 40X water objective and a DPSS 561 nm laser to visualize the phalloidin-stained F-actin filaments.

### RNA Extraction and RT-qPCR

At days 1, 7 and 21 after fabrication, hydrogels were washed in PBS 1X. Following the protocol of Bougault et al. (2009), hydrogels were dissolved in solubilization buffer (1.5 mL QG buffer (19063, Qiagen), 2 mL RLT lysis buffer (79216, Qiagen), 20 μL 2-mercaptoethanol) and RNA was extracted using the RNeasy Mini Kit (74106, Qiagen). RNA was eluted in 30 μL RNase-free water and the RNA yield and purity were assessed with a NanoDrop 1000 Spectrophotometer (Thermo Fisher Scientific). RNA (100 ng to 1 μg) was reverse-transcribed into cDNA in a 60 μL volume using the TaqMan Reverse Transcription kit (N8080234, Applied Biosystems). For each qPCR reaction, 10 ng of cDNA was mixed with TaqMan primers (COL1A2: Bt03214883_m1; COL2A1: Bt03251861_m1; ACAN: Bt03212186_m1; YWHAZ: Bt01122444_g1), TaqMan Fast Universal PCR Master Mix (2X) (4352042, Applied Biosystems) and RNAse-free water to a total volume of 10 μL. Gene expression was measured in duplicates with the CFX96 Touch Detection System (Bio-Rad). YWHAZ was used as a reference gene. Results are shown as 2^–ΔCt^ values (i.e., relative to YWHAZ).

### GAG and DNA Quantification

At days 1, 7 and 21 after fabrication, hydrogels were snap-frozen in liquid nitrogen and lyophilized overnight. Hydrogels and GAG standards (bovine chondroitin sulfate, C6737, Sigma-Aldrich) ranging from 0.78 to 50 μg/mL were digested for 16 h at 60°C in 140 μg/mL papain in 100 mM phosphate buffer pH 6.5, 5 mM L-cysteineHCl and 5 mM Na_2_EDTA. After digestion, the samples and the standards were centrifuged for 10 min at 12’000 rpm and 40 μL of the supernatant were pipetted in duplicate in a 96-well plate. Dimethylmethylene blue solution (Farndale et al., 1986) was added (150 μL/well) and absorbance was measured immediately at 540 nm with a reference at 595 nm using a plate reader (Infinite M200 PRO, TECAN). The Quant-iT PicoGreen^TM^ dsDNA Assay Kit (P7589, Thermo Fisher Scientific) was used for DNA quantification according to the manufacturer protocol. For each sample, the GAG quantity (μg) was normalized to the DNA quantity (μg).

### 2D Cell Adhesion Assay

Hydrogel solutions were prepared as described above and 10 μL were cast in a 96-well angiogenesis plate (89646, ibidi). Bovine NP cells were seeded on top of the hydrogels at a density of 60’000 cells/cm^2^ in 50 μL/well culture medium and incubated overnight at 37°C, 5% CO_2_. The next day, hydrogels were washed three times with PBS 1X, fixed and stained with Alexa-Fluor 568 phalloidin and DAPI as described above. A confocal microscope (Leica TCS SP8) with 10 and 20X dry objectives and a DPSS 561 nm laser was used to image the cells on the hydrogels. Automated counting of the DAPI-stained cells was performed with the ImageJ software.

### Cell-Laden Hydrogel Dynamic Compression

Agarose and 2 mg/mL agarose-collagen hydrogels were pre-cultured in the silicon rings used for molding for 7 days at 37°C, 5% CO_2_, in DMEM/F-12 without phenol red (11039-021, Gibco) supplemented with 10% FCS and 0.1% ampicillin (A0839, AppliChem). Two hours before compression, the medium was replaced with serum-free medium. For dynamic compression, the hydrogels in silicon rings were transferred to the wells of a commercial compression device (MCTR, CellScale) in 1 mL of serum-free medium. Hydrogels were dynamically compressed for 5, 10 or 20 min with a sine wave function at 0.5 Hz with a nominal force of 73 and 3 N of pre-load (corresponding to a 20% strain ratio) at 37°C, 5% CO_2_. Non-compressed hydrogels were kept in identical conditions in a 12-well plate. Immediately after mechanical loading, hydrogels were processed for protein analysis.

### Protein Extraction and Western Blot

Hydrogels were cut into four pieces, washed with PBS 1X, snap-frozen in liquid nitrogen and lyophilized overnight. RIPA buffer supplemented with protease and phosphatase inhibitor cocktails (78430; 78428, Thermo Fisher Scientific) was used to lyse the hydrogels on ice (100 μL/gel) for 1 h. The lysates and the soaked hydrogels were then transferred to NucleoSpin filter columns (740606, Macherey-Nagel) and centrifuged for 1 h at 4°C at 12’000 rpm. For all samples, 22.5 μL of lysate were mixed with 4X Laemmli buffer (1610747, Bio-Rad), heated for 5 min at 95°C, and loaded onto a 4–20% gradient gel (4568093, Bio-Rad). Proteins were separated by electrophoresis in a Mini-PROTEAN Tetra Cell (Bio-Rad) and transferred to a PVDF membrane (1704156, Bio-Rad). The membrane was washed 3 × 10 min with Tris Buffered Saline with 0.05% TWEEN 20 (TBS-T) and blocked for 2 h at RT with 5% skim milk diluted in TBS-T. Primary antibodies (FAK: 3285; phospho-FAK (Tyr397): 3283; β-tubulin: 2146; Cell Signaling Technology) were applied 1:1000 in 3% BSA in TBS-T overnight at 4°C on a rocker. The next day, the membrane was washed 3 × 10 min with TBS-T and incubated for 1 h at RT on a rocker with the secondary antibody (anti-rabbit IgG HRP: 7074, Cell Signaling Technology) diluted 1:1000 in 5% skim milk in TBS-T. After washing 3 × 10 min with TBS-T, proteins were detected with a chemiluminescence substrate (34076, Thermo Fisher Scientific) on a ChemiDocTouch Imaging System (Bio-Rad). The density of the bands was semi-quantified using the Volume Tools of the ImageLab software (Bio-Rad) and by fitting a rectangular shape of same area to each band. For each blot, the density of each band was normalized by the one of the agarose non-compressed control. Phospho-FAK and FAK were each expressed relative to the β-tubulin loading control and the pFAK/FAK ratio was calculated.

### Statistical Analysis

For each experimental condition, cells from 3 to 4 different animals were used. Cell-free experiments were conducted in triplicates. Data consistency was checked and data were screened for outliers and normal, Gamma, Log-normal and Tweedie distributions by using quantile plots. Continuous variables were also tested for these distributions by using Kolmogorov–Smirnov test. Due to the small sample size, independent and dependent bootstrap-*t*-tests were used based on 5000 Monte Carlo simulations. Additionally, generalized estimation equation models (GEE) were used to analyze data and corresponding LSD tests for pairwise comparisons. Independent and unstructured working correlation matrices were used for modeling. All reported tests were two-sided, and *p*-values < 0.05 were considered as statistically significant. Results are shown as means ± standard deviation (SD). All statistical analyses were performed with STATISTICA 13 (Hill, T. & Lewicki, P. Statistics: Methods and Applications. StatSoft, Tulsa, OK, United States) and PASW 21 (IBM SPSS Statistics for Windows, Version 21.0., Armonk, NY, United States).

## Results

### Hydrogel Homogeneity

**FIGURE 1:**
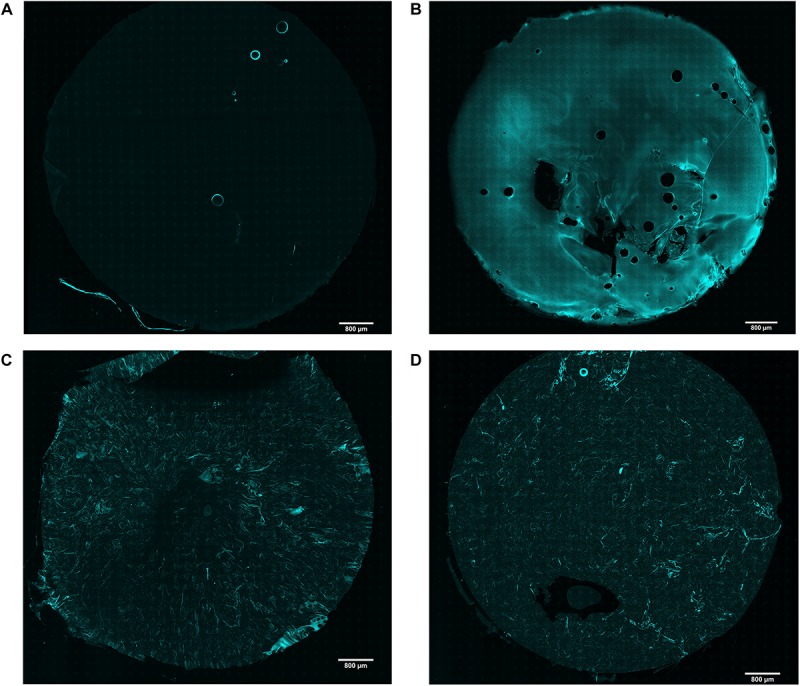
Representative images of confocal reflection microscopy of collagen I in **(A)** agarose; **(B)** collagen; **(C)** agarose-collagen 2 mg/mL; and **(D)** agarose-collagen 4.5 mg/mL hydrogels. Scale bar = 800 μm.

Although agarose and collagen I are both hydrophilic materials, it is challenging to mix them homogenously due to their different gelling temperatures and mechanisms. While low gelling temperature agarose is liquid above 65°C and gels at around 26–30°C, collagen I is liquid at 4°C and gels at 37°C. It was therefore crucial to first test whether mixing by vortexing produced a homogenous distribution of these materials. As expected, reflection microscopy of collagen I revealed no signal in agarose hydrogels (Figure 1A), while a strong signal was detected in collagen hydrogels (Figure 1B). In comparison, both the 2 and 4.5 mg/mL agarose-collagen hydrogels displayed an intermediate signal, with collagen I fibrillar structures appearing homogenously distributed throughout the agarose matrix (Figures 1C,D).

### Hydrogel Rheological Properties

**FIGURE 2:**
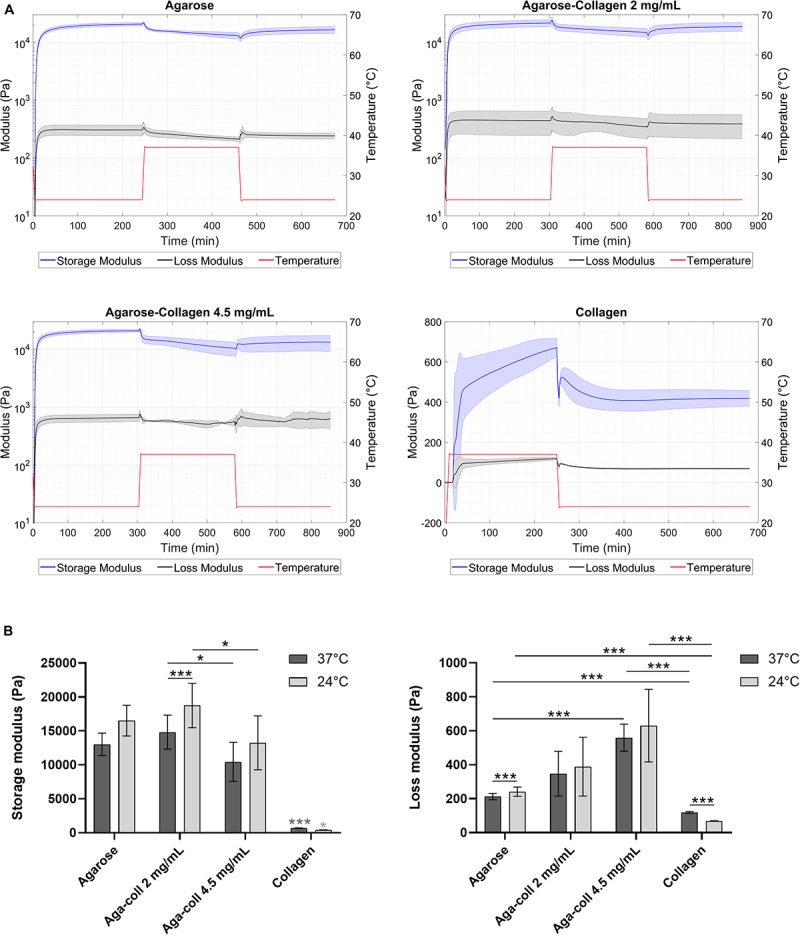
**(A)** Rheological properties of agarose, agarose-collagen 2 mg/mL, agarose-collagen 4.5 mg/mL, and collagen hydrogels with temperature variation over time. **(B)** Extracted storage and loss moduli at 37 and 24°C. *n* = 3 independent replicates, mean ± SD; *p < 0.05, ***p < 0.001; light gray asterisks on bars indicate a statistically significant difference compared to all other hydrogel types at the same temperature.

Gel formation of agarose and agarose-collagen 2 and 4.5 mg/mL hydrogels started immediately after lowering the temperature to 24°C. The storage modulus (G’) reached a plateau at 20.7 ± 0.3, 21.6 ± 0.4, and 20.8 ± 0.7 kPa, respectively, after 240 or 300 min (Figure 2A). This gelation process is governed by the formation of hydrogen bonds resulting in the aggregation of double helices (Velasco et al., 2012). To promote collagen fibril formation, the temperature was then increased to 37°C, at which G’ dropped to 13.0 ± 0.2, 14.8 ± 0.3, and 10.5 ± 0.6 kPa, respectively (Figure 2A). The temperature was subsequently decreased again to 24°C in order to evaluate the rheological properties of the formed hydrogels at room temperature. At this final 24°C temperature, G’ increased to 16.5 ± 0.2, 18.8 ± 0.4 and 13.3 ± 0.6 kPa for the agarose, agarose-collagen 2 and 4.5 mg/mL hydrogels, respectively (Figure 2A). The loss modulus (G”) of the agarose-based hydrogels varied with temperature similarly to the G’, but displayed values about one order of magnitude lower (Figure 2A). This highlights the mainly elastic nature of the agarose-based hydrogels.

Formation of collagen hydrogels started after increasing the temperature to 37°C. The G’ reached a plateau at 0.7 ± 0.1 kPa after 240 min (Figure 2A). Upon decrease of the temperature to 24°C, a decrease in G’ to 0.4 ± 0.1 kPa was observed (Figure 2A).

Collagen hydrogels were significantly softer than agarose or agarose-collagen hydrogels at both 37 and 24°C (Figure 2B). While agarose and agarose-collagen 2 mg/mL hydrogels had a similar G’ at both 37 and 24°C, agarose-collagen 4.5 mg/mL hydrogels displayed a significantly lower G’ compared to agarose-collagen 2 mg/mL hydrogels at both temperatures (Figure 2B). Additionally, the G” of agarose-collagen 4.5 mg/mL hydrogels was significantly higher compared to agarose hydrogels at 37°C (Figure 2B).

### Hydrogel Dimensional Stability

**FIGURE 3:**
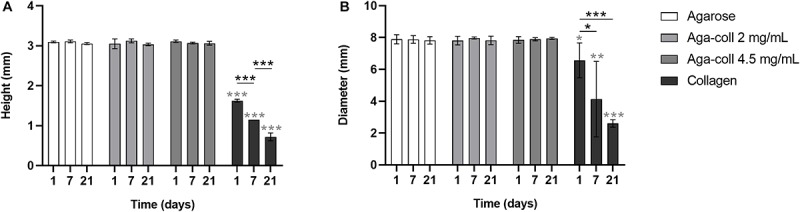
**(A)** Height and **(B)** diameter of agarose, agarose-collagen 2 mg/mL, agarose-collagen 4.5 mg/mL, and collagen hydrogels at days 1, 7, and 21 after fabrication. *n* = 3 biological replicates, mean ± SD; *p < 0.05, **p < 0.01, ***p < 0.001; light gray asterisks on bars indicate a statistically significant difference compared to all other hydrogel types at the same day.

Another important feature of biomaterials intended for dynamic compression, and mechanobiology studies in general, is size stability. Cell-laden agarose-based hydrogels showed unaltered height and diameter at days 1, 7 and 21 (Figures 3A,B). In comparison, collagen hydrogels displayed compaction, with a significantly reduced height and diameter at all time-points (Figures 3A,B). Within the collagen group, hydrogels significantly shrunk over time in both height and diameter (Figures 3A,B).

### Cell Viability and Proliferation

**FIGURE 4:**
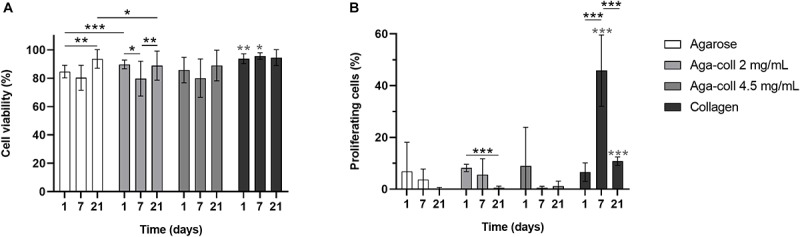
**(A)** Cell viability percentage and **(B)** percentage of proliferating cells of agarose, agarose-collagen 2 mg/mL, agarose-collagen 4.5 mg/mL, and collagen hydrogels at days 1, 7, and 21 after fabrication. *n* = 3 biological replicates, mean ± SD; *p < 0.05, **p < 0.01, ***p < 0.001; light gray asterisks on bars indicate a statistically significant difference compared to all other hydrogel types at the same day.

As cells were briefly subjected to mechanical and thermal stress during hydrogel fabrication due to vortexing and non-physiological temperatures, cell viability was tested. Viability ranged from 80 to 95% and was slightly higher for collagen hydrogels at days 1 and 7 (Figure 4A). The cell viability in agarose-based hydrogels marginally decreased at day 7, then increased at day 21 (Figure 4A).

The proliferation rate of IVD cells *in vivo* is relatively low, with only 4–5% cells positively stained for Ki67 in human samples (Gruber et al., 2009). A high proliferation rate is therefore undesired, as it might promote cell de-differentiation. The percentage of cells in the S phase was generally variable and low (<10%) in all experimental groups, except for collagen hydrogels at day 7, which displayed a significantly higher proliferation percentage of 45.8% (Figure 4B). The proliferation rate of agarose-collagen 2 mg/mL decreased between days 1 and 21 (Figure 4B).

### 3D Cell Morphology

**FIGURE 5:**
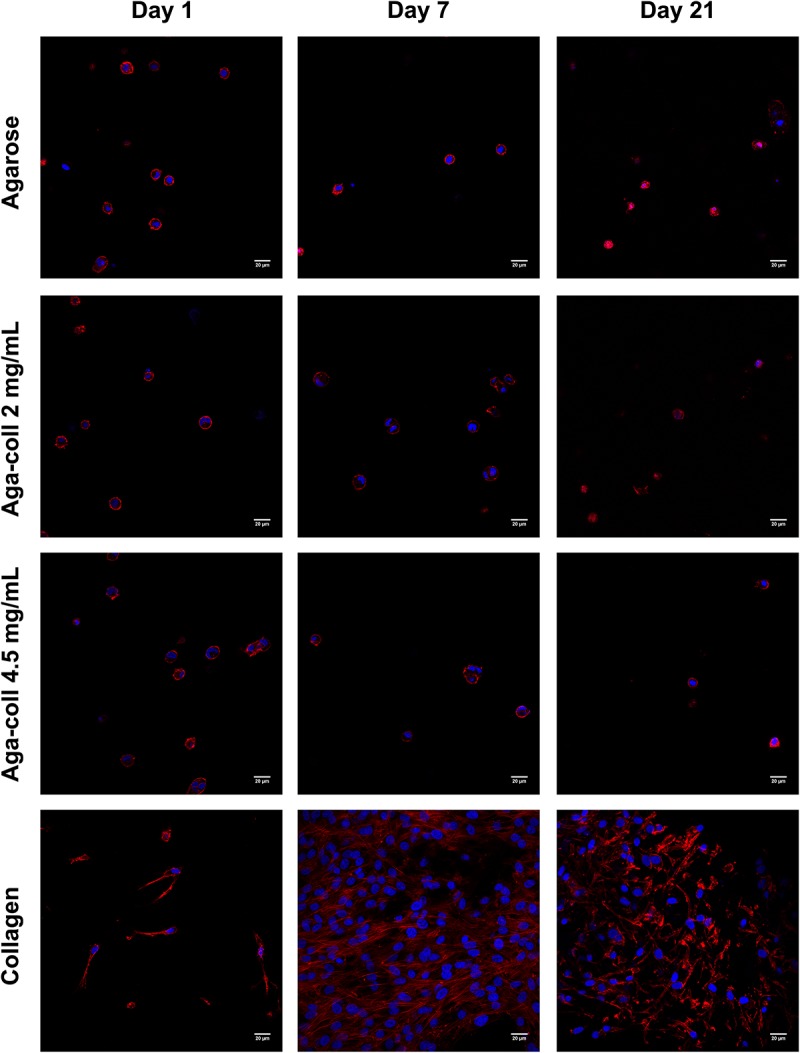
Representative images of cell morphology (red = phalloidin, blue = DAPI) of agarose, agarose-collagen 2 mg/mL, agarose-collagen 4.5 mg/mL, and collagen hydrogels at days 1, 7 and 21 after fabrication. Scale bar = 20 μm.

In order to understand the interaction between cells and the different hydrogels, cells encapsulated in 3D hydrogels were stained and imaged over time. The 3D cell morphology in all agarose-based hydrogels remained round at all time-points, while cells in collagen hydrogels displayed an elongated morphology and a higher cell density at days 7 and 21 (Figure 5).

### ECM Gene Expression and GAG Production

**FIGURE 6:**
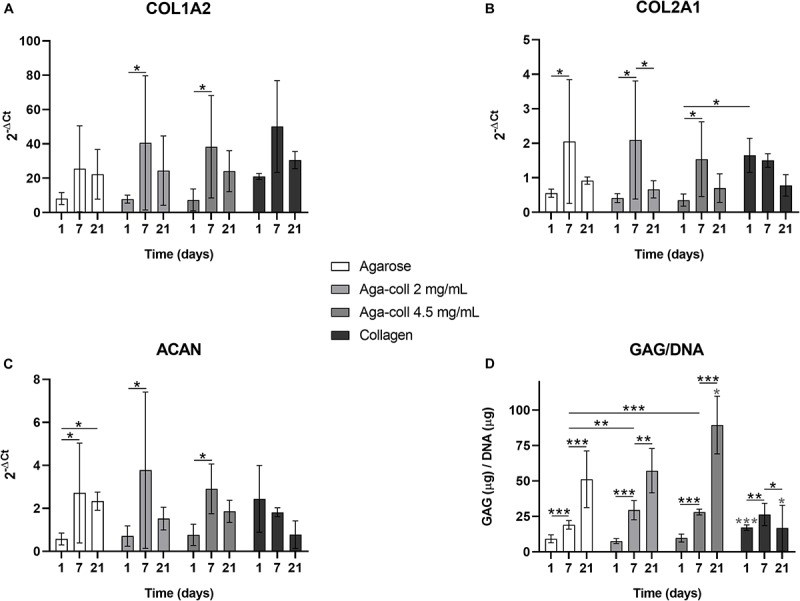
Gene expression of **(A)** COL1A2, **(B)** COL2A1, **(C)** ACAN, and **(D)** GAG/DNA quantification of agarose, agarose-collagen 2 mg/mL, agarose-collagen 4.5 mg/mL, and collagen hydrogels at days 1, 7, and 21 after fabrication. *n* = 3 biological replicates, mean ± SD; *p < 0.05, **p < 0.01, ***p < 0.001; light gray asterisks on bars indicate a statistically significant difference compared to all other hydrogel types at the same day.

We investigated the mRNA expression of collagen I (COL1A2), collagen II (COL2A1) and aggrecan (ACAN), as the main components of the ECM of the IVD. The gene expression of COL1A2, COL2A1 and ACAN significantly increased between days 1 and 7 in composite agarose-collagen hydrogels at both collagen concentrations (Figures 6A–C). This change was also noted for COL2A1 and ACAN in agarose hydrogels (Figures 6B,C). ACAN was additionally significantly increased between days 1 and 21 in agarose hydrogels (Figure 6C). This increase over time was confirmed when measuring the GAG/DNA content. At day 1, the GAG/DNA ratio was significantly higher in collagen hydrogels compared to other gels (Figure 6D). Nevertheless, an increase over time (between day 1 and day 7, and between day 7 and day 21) was observed in all agarose-based hydrogels, which finally surpassed collagen hydrogels at day 21 (Figure 6D). Interestingly, the GAG/DNA content was significantly higher in both blended agarose-collagen hydrogels compared to the agarose group at day 7 (Figure 6D).

### 2D Cell Adhesion

**FIGURE 7:**
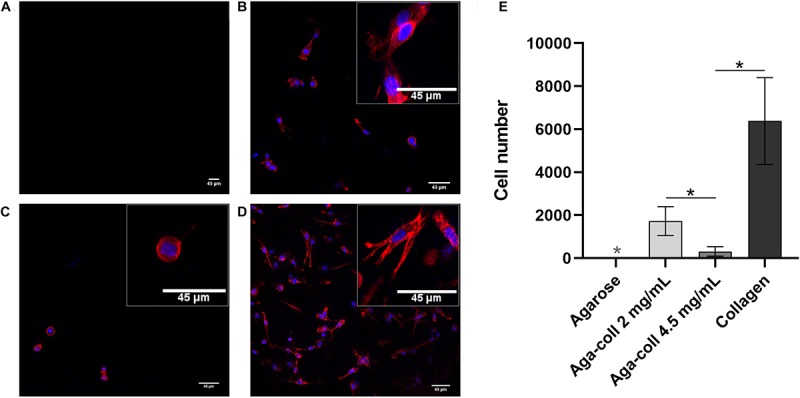
Representative images and enlarged insets of cells (red = phalloidin, blue = DAPI) seeded on **(A)** agarose, **(B)** agarose-collagen 2 mg/mL, **(C)** agarose-collagen 4.5 mg/mL, and **(D)** collagen 2D hydrogels at day 1 after fabrication. Scale bars = 45 μm. **(E)** Cell count per hydrogel type. *n* = 3 biological replicates, mean ± SD; *p < 0.05; the light gray asterisks on the agarose bar indicates a statistically significant difference compared to all other hydrogel types.

As expected, no cells could adhere to agarose 2D hydrogels (Figures 7A,E). In comparison, all substrates containing collagen displayed a significantly higher number of cells (Figures 7B–D): 1’728 ± 671 cells on the 2 mg/mL agarose-collagen gels; 314 ± 220 on the 4.5 mg/mL agarose-collagen gels; and 6’381 ± 2017 cells on the collagen gels (Figure 7E). Surprisingly, the number of cells adhering to the 4.5 mg/mL agarose-collagen gels was significantly reduced compared to the 2 mg/mL and the collagen groups (Figure 7E). The morphology of the cells seeded on the 4.5 mg/mL agarose-collagen hydrogels was also slightly rounder (inset of Figure 7C) compared to the more elongated polygonal shape of cells seeded on 2 mg/mL agarose-collagen and collagen hydrogels (insets of Figures 7B,D).

### FAK Phosphorylation in Dynamic Compression

**FIGURE 8:**
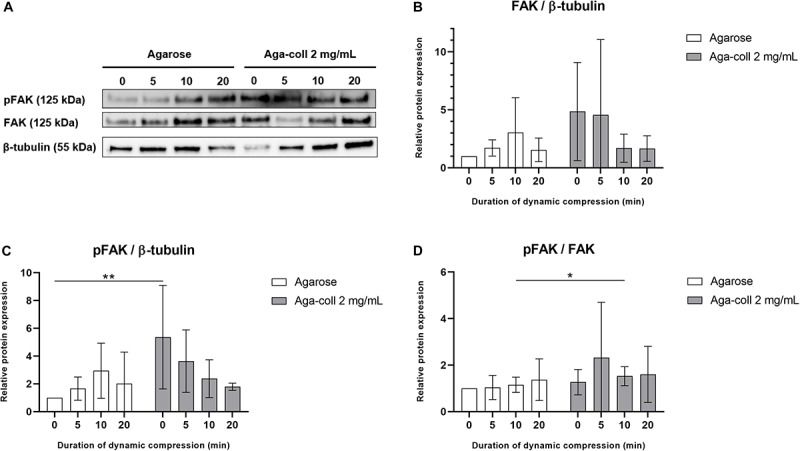
**(A)** Western blot of agarose and agarose-collagen 2 mg/mL hydrogels dynamically compressed for 0, 5, 10, or 20 min. Densitometry analysis of **(B)** relative FAK, **(C)** pFAK, and **(D)** pFAK/FAK protein expression, *n* = 4 biological replicates, mean ± SD; *p < 0.05, **p < 0.01.

Based on their superior mechanical and adhesive properties, the agarose-collagen 2 mg/mL hydrogels were selected to test the capacity to transmit mechanical load to cells. Agarose and 2 mg/mL agarose-collagen gels were pre-cultured for 7 days and FAK expression and phosphorylation was semi-quantified after hydrogel dynamic compression for 0, 5, 10, or 20 min (Figure 8A). No statistically significant difference was found across conditions in the expression of FAK relative to β-tubulin (Figure 8B). Both hydrogel types did not show any significant change in FAK phosphorylation between compressed and non-compressed samples (Figures 8C,D). However, pFAK levels relative to β-tubulin were considerably higher (5.36-fold) in 2 mg/mL agarose-collagen gels compared to agarose gels in non-compressed samples (Figure 8C). The composite gels further displayed a slight but significant increase in pFAK/FAK values compared to the agarose gels 10 min after compression (Figure 8D).

## Discussion

Biomaterials combining strong and tunable mechanical properties with the biological cues of natural ECM proteins are in demand for mechanotransduction studies. This study aimed to introduce agarose-collagen composite hydrogels as a simple, inexpensive, and effective option in the context of IVD mechanobiology investigations. While the typical agarose concentration for dynamic compression studies of 2% wt/vol was kept constant, collagen I was physically blended into agarose at two final concentrations of 2 and 4.5 mg/mL. Reflection microscopy showed that mixing by vortexing the two polymers was an effective method to achieve a homogenous distribution of collagen throughout the gels. Previous studies involving scanning electron microscopy observed the formation of an intricate network between the two materials, where fine “web-like” agarose intercalated between larger entangled collagen fibers (Ulrich et al., 2010; Lake and Barocas, 2011). While adding collagen at 4.5 mg/mL decreased the elasticity and increased the viscosity of the hydrogels, blending 2 mg/mL collagen did not affect the rheological properties of the gels. The blending of collagen I at the higher concentration might impair the formation of hydrogen bonds in agarose and the resulting formation and aggregation of double helices that govern agarose gelling mechanism. The collagen fibers might thus create macroscopic defects in the agarose non-fibrillar matrix. This might also explain why the decrease in storage modulus of the 4.5 mg/mL hydrogels was only observed after the hydrogel was heated to 37°C, which triggered the formation of collagen fibers. Despite the introduction of degradable collagen I, agarose-collagen hydrogels presented suitable dimensional stability, contrary to collagen hydrogels that underwent cell-mediated contraction (Bell et al., 1979). This feature represents an advantage, especially in the case where these hydrogels need to be subjected to mechanical loading with a displacement-controlled bioreactor. NP cells were viable and their proliferation was low in all agarose-based hydrogels, approaching proliferation rates of IVD cells *in vivo*. Interestingly, the presence of collagen did not alter the round 3D morphology of cells embedded in blended hydrogels. This suggests a dense matrix mainly composed of agarose. The gene expression of collagen I and II, as well as aggrecan increased at day 7 in blended hydrogels, and the GAG/DNA content was also increased compared the agarose hydrogels at this time-point. A similar increase in GAG/DNA was also observed in chondrocyte-laden agarose-poly(ethylene glycol) diacrylate (PEGDA) interpenetrating networks when aggrecan was physically blended in Ingavle et al. (2014). While collagen I blending increased cell adhesion, the effect was surprisingly stronger for the lower concentration of collagen at 2 mg/mL. We attribute this finding to the difference in mechanical properties between the 2 and 4.5 mg/mL hydrogels. In this case, the lower stiffness of the 4.5 mg/mL hydrogels might over-rule their increased adhesion ligand density, thus resulting in reduced cell adhesion. This phenomenon suggests a non-linear response, similar to the one observed for the spread area of cells on gels (Discher et al., 2005). This stiffness-dependent cell adhesion behavior was already observed with human dermal fibroblasts seeded on laminin peptide-agarose gels in the study of Yamada et al. (2012). Enhanced cell adhesion due to collagen blending at 2 mg/mL was further confirmed by higher levels of FAK phosphorylation relative to β-tubulin in non-compressed samples, and higher pFAK/FAK values 10 min after compression. While FAK activation is usually associated with cell seeding on stiff 2D substrates (Lee et al., 2019), little is known about the effect of stiffness in 3D materials. No significant difference in mechanical properties was observed between agarose and 2 mg/mL agarose-collagen gels. Therefore, FAK phosphorylation was likely induced by the interaction of cells with the collagen present in the matrix via integrin receptors (Lee et al., 2019). The fact that dynamic compression of the hydrogels did not influence the level of FAK phosphorylation additionally suggests an effect inherently driven by the composite biomaterial. In fact, the fibrillar nature of the collagen introduced in the composite network might guide the promotion of focal adhesion formation, as this phenomenon has only been shown in fibrillar 3D scaffolds so far (Cukierman et al., 2001; Doyle et al., 2009; Nam et al., 2016). Recently, it has been shown that focal adhesion formation plays an important role in the response of NP cells to their surrounding matrix and the presence or absence of integrin binding sites (Krouwels et al., 2018).

Our agarose-collagen gels composed of 2% wt/vol agarose and 2 mg/mL collagen contain approximatively 10% fibrillar collagen (collagen/agarose wt/wt), thus mimicking the NP matrix well (Lake and Barocas, 2011). However, the ratio of collagen and agarose can be tuned in order to mimic other native tissues such as ligaments, tendons and cartilage. On the biological level, IVDs and cartilage share several characteristics such as a relatively stiff ECM, high collagen II and proteoglycan content, low proliferation and round cell morphology. The use of blended agarose-collagen could therefore be extended to the embedding of chondrocytes and the study of their mechanotransduction mechanisms. Such studies would be beneficial to shed light on the pathways leading to osteoarthritis, one of the great health challenges of the increasingly aging population. With a similar focus, blended agarose-collagen hydrogels might constitute promising substrates for mesenchymal stem cells chondrogenic differentiation.

## Conclusion

Agarose-collagen blended hydrogels are easy to produce, inexpensive and do not require cytotoxic reagents. We showed that they constitute a valid 3D culture model for dynamic compression and investigation of IVD cell mechanotransduction. Using physical blending, a homogenous interconnected network was formed, presenting both the biofunctionality of collagen I and the mechanical strength and stability of agarose. In fact, the addition of collagen type I at 2 mg/mL conserved many qualities of agarose: non-degradability, strong elastic properties, ability to promote ECM gene expression, and low proliferation and round morphology of embedded cells. Furthermore, blended agarose-collagen hydrogels not only enhanced the production of GAG at day 7 compared to agarose gels, but also increased cell adhesion and FAK activation, one of the first steps in the integrin-mediated mechanotransduction mechanisms. In light of the described findings, agarose-collagen hydrogels represent an improved alternative to agarose gels for the exploration of cell-matrix interaction and mechanotransduction mechanisms of native tissues constituted of a non-fibrillar matrix (such as proteoglycans and GAGs) and collagens. In this context, they could further be used to explore stem cell chondrogenic differentiation.

## Data Availability Statement

All datasets generated for this study are included in the article/supplementary material.

## Author Contributions

EC conceived the project, designed and conducted experiments, analyzed data, and wrote the manuscript. SB and SH contributed equally and developed protocols and conducted experiments. PF helped developing protocols for rheological measurements. WH performed statistical analysis. SF and KW-K provided funding and supervised the project. All authors reviewed the manuscript.

## Conflict of Interest

The authors declare that the research was conducted in the absence of any commercial or financial relationships that could be construed as a potential conflict of interest.
